# Polarimetric imaging for cervical pre-cancer screening aided by machine learning: *ex vivo* studies

**DOI:** 10.1117/1.JBO.28.10.102904

**Published:** 2023-07-06

**Authors:** Demelza Robinson, Kevin Hoong, Willem Bastiaan Kleijn, Alexander Doronin, Jean Rehbinder, Jeremy Vizet, Angelo Pierangelo, Tatiana Novikova

**Affiliations:** a Victoria University of Wellington, School of Engineering and Computer Science, Wellington, New Zealand; b Universite de Strasbourg, Laboratoire ICube, Illkirch, France; c ArianeGroup, Les Mureaux, France; d Institut Polytechnique de Paris, Ecole Polytechnique, CNRS, LPICM, Palaiseau, France; e Florida International University, Department of Biomedical Engineering, Miami, Florida, United States

**Keywords:** Mueller polarimetry, cervical cancer, optical diagnostics, machine learning

## Abstract

**Significance:**

Wide-field imaging Mueller polarimetry is an optical imaging technique that has great potential to become a reliable, fast, non-contact *in vivo* imaging modality for the early detection of, e.g., deceases and tissue structural malformations, such as cervical intraepithelial neoplasia, in both clinical and low-resource settings. On the other hand, machine learning methods have established themselves as a superior solution in image classification and regression tasks. We combine Mueller polarimetry and machine learning, critically assess the data/classification pipeline, investigate the bias arising from training strategies, and demonstrate how higher levels of detection accuracy can be achieved.

**Aim:**

We aim to automate/assist with diagnostic segmentation of polarimetric images of uterine cervix specimens.

**Approach:**

A comprehensive capture-to-classification pipeline is developed in house. Specimens are acquired and measured with imaging Mueller polarimeter and undergo histopathological classification. Subsequently, a labeled dataset is created within tagged regions of either healthy or neoplastic cervical tissues. Several machine learning methods are trained utilizing different training-test-set-split strategies, and their corresponding accuracies are compared.

**Results:**

Our results include robust measurements of model performance with two approaches: a 90:10 training–test-set-split and leave-one-out cross-validation. By comparing the classifier’s accuracy directly with the ground truth obtained during histology analysis, we demonstrate how conventionally used shuffled split leads to an over-estimate of true classifier performance (0.964±0.00). The leave-one-out cross-validation, however, leads to more accurate performance (0.812±0.21) with respect to newly obtained samples that were not used to train the models.

**Conclusions:**

Combination of Mueller polarimetry and machine learning is a powerful tool for the task of screening for pre-cancerous conditions in cervical tissue sections. Nevertheless, there is a inherent bias with conventional processes that can be addressed using more conservative classifier training approaches. This results in overall improvements of the sensitivity and specificity of the developed techniques for “unseen” images.

## 1 Introduction

Cervical cancer is typically preceded by the growth of abnormal cells in the epithelial lining of the uterine cervix. If these are detected early enough, treatment can prevent their progression into malignancy. Human papillomavirus (HPV) is implicated in the majority of cervical cancer cases.^1^ However, despite the availability of the HPV vaccine, cervical cancer persists as a challenging health problem worldwide.^2^ Late diagnosis^3^ and HPV vaccination status^4^ constitute two of the most significant risk factors in cervical cancer mortality. This coincides with the highest incidence and mortality rates occurring in developing, low-income countries,^5^ where screening and vaccination resources can be greatly limited. Early detection of cervical intraepithelial neoplasia (CIN), or pre-cancerous alterations of cervical tissue, is constrained by the fact that these changes are difficult to visualize with the naked eye.^6^ In developed countries, the standard screening for cervical pre-cancer includes cytopathological Pap test followed by the visual examination of the cervix with a colposcope, if necessary.^7^ The latter is done after the application of contrast enhancing agents (acetic acid, iodine Lugol’s solution). Then, the biopsies are taken from the acetic acid-positive and iodine-negative zones. When the presence of malignant cells is confirmed by a pathologist, the abnormal zones are surgically removed. However, the accuracy of the colposcopy diagnostic step is strongly affected by the training and experience of medical doctors. Developments in this screening process could permit earlier detection of cervical cancer worldwide, in turn making it more accessible to developing nations.

In recent decades, considerable advancements have been made in the development of optical techniques applicable to CIN detection. Nevertheless, high-quality optical diagnostics of the internal structure and functional malformations within biological tissues is significantly impeded due to multiple scattering of light. A number of theoretical and experimental techniques have been developed to assess this unique phenomenon.^8^^,^^9^ Diffuse optical spectroscopy, non-linear Raman spectroscopy, optical coherence tomography, and confocal microscopy all exhibit promising results for visualizing the precursory cell abnormalities that are characteristic of CIN. The high spatial resolution that these methods provide, however, is limited by a small field of view that still requires the time-consuming inspection of a sample by pathologists.^7^ Producing wide-field cervical images with high-contrast CIN zones is essential for a reliable screening process. These properties enable delineation of precise borders of the pre-cancerous tissue regions. Greater confidence in CIN boundaries can permit a more informed excision of a high-grade CIN3 lesion.^7^ It is imperative that highly precise boundaries are formed, as being too conservative can leave pre-cancerous cells *in situ*, which can develop into malignancies and metastasise, whereas excising too much can impair normal cervical tissue function.^10^

Polarization or spin angular momentum (SAM) is one of light’s most salient features, along with spectral and coherence properties.^11^^,^^12^ It has been previously established that light polarization is extremely sensitive to the micro-architecture and optical parameters of a variety of studied media. This property has been extensively explored in a range of contexts including remote sensing,^13^^–^^15^ optical metrology,^16^^–^^18^ and biomedical diagnostics.^19^^–^^21^ Our previous studies clearly demonstrated that simple (i.e., circularly) polarized light is able to distinguish different grades of cancer.^22^ This motivated an investigation into the application of a promising wide-field polarimetric technique, namely Mueller matrix (MM) polarimetry.^23^^,^^24^ This technique has great potential in becoming a reliable, fast (the acquisition of 16 MM images in <1  s was demonstrated in Ref. 25), non-contact *in vivo* imaging modality for the early detection of CIN in both clinical and low-resource settings.^26^ The wide-field imaging MM polarimeter generates a 4×4 matrix of images of a sample. Each of the 16 images captures changes in the polarization of light back-scattered by a sample, with each corresponding to a different polarization setting of an incident light beam.^27^^,^^28^ The optical properties of a sample are encoded in the 16 elements of its MM, but there is no straightforward interpretation of this matrix with regards to identifying markers of CIN. Polarimetric properties of a sample (e.g., diattenuation, retardance, and depolarization) are calculated from the experimental MM by applying one of the available non-linear, pixel-wise data compression algorithms.^20^^,^^29^^,^^30^ It has been demonstrated that several optical properties, including both depolarization and scalar retardance values, are able to distinguish between regions corresponding to CIN and those belonging to healthy cervical tissue.^31^ Several statistical and machine learning (ML) algorithms have been developed and tested for the diagnostic segmentation of polarimetric images of uterine cervix and accurate detection of CIN zones.^32^^–^^34^ An investigation combining wide-field imaging MM polarimetry and ML techniques for detection of cervical pre-cancer has yet to be undertaken.

In this work, we evaluate various ML methods to carry out CIN diagnosis using the MM images of 23 formalin fixed cervical specimens acquired in the framework of the PAIR Gynéco project funded by the National Institute of Cancer in France.^31^ Each of these specimens was examined by pathologists and has a histologically confirmed CIN diagnosis. The diagnosis is provided in the form of a spatial mask, one for each sample, which can be overlaid onto each of the 16 elements of the MM. The mask indicates which regions were classified as CIN and which were classified as healthy. Masked features were extracted and used to train classifiers for the task of distinguishing pixels as belonging to CIN or healthy tissue. Only some regions of each sample could be classified with confidence by the pathologists. The pixels with labels were used exclusively in the supervised training process because the other pixels have no associated classification to learn from.

This paper is organized as follows. Section 2 details the related background for this study, including a description of the data collection methods and the ML techniques used for classification of CIN and healthy tissue. Section 3 presents the proposed capture-to-classification pipeline and details of the experimental design. Section 4 describes the classification results across a variety of ML classifiers. Finally, Sec. 5 covers the conclusions and directions for future work.

## 2 Background and Related Work

### 2.1 Multi-Spectral Wide-Field Imaging MM Polarimeter

**Fig. 1:**
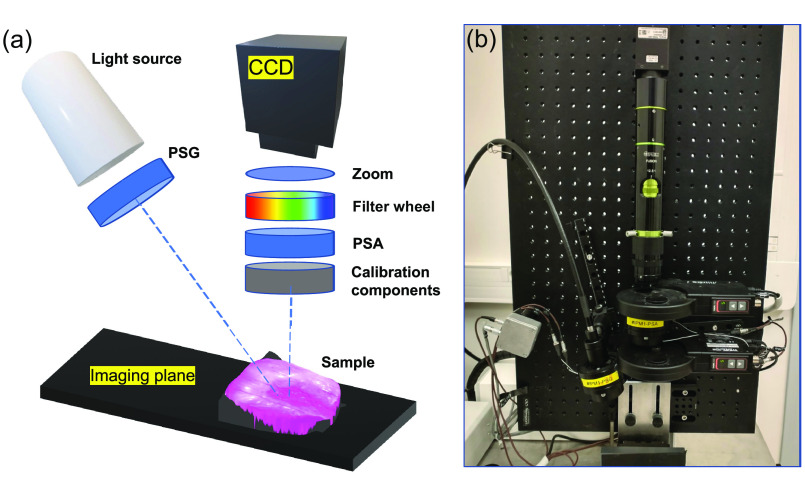
(a) Schematic presentation and (b) the actual photograph of the experimental setup used to acquire MM images.

Conization specimens were imaged using the full-field multi-spectral Mueller polarimetric imaging system in a backscattering configuration described in Refs. 31, 33, 34. The main components and operational principle of the optical setup are briefly recalled in this section. Figure 1 shows the schematic layout of the multi-spectral wide-field imaging MM polarimeter used for the data acquisition. Polarization modulation of incident light from a broad band white light source is carried out by the polarization state generator (PSG), which includes a sequential assembly of a linear polarizer and two voltage-driven ferroelectric liquid crystals (FLCs). The polarization state analyzer (PSA) inserted in the detection arm of the instrument contains the same optical components as the PSG but assembled in the reverse order. Each FLC operates in transmission as a quarter wave plate with the orientation of the fast optical axis switching between 0 deg and 45 deg in the laboratory reference framework depending on the applied voltage. A light-emitting diode was used as the source of incoherent white light.

The incident light beam illuminates a sample at an incidence angle of ∼15  deg, producing a spot size of ∼10  cm along the main ellipse axis within the imaging plane. Back-scattered light from a sample passes through the PSA and rotating wheel that contains 40 nm band-pass interference filters enabling the measurements from 450 to 700 nm in 50 nm increments. The image is recorded with a 600×800  pixels CCD camera (Stingray F080B, Allied Vision, Germany) placed normal to the imaging plane. To measure all 16 elements of MM, one needs to perform at least 16 intensity measurements. In our polarimetric imaging system, four different polarization states of incident light beam are produced sequentially by the PSG and projected onto four different polarization configurations of the PSA, thus resulting in 4×4=16 intensity images detected by the camera. The Eigenvalue Calibration Method by E. Compain^35^ was used to calibrate the instrument, with the reference samples (two crossed polarizers and a wave plate at 30 deg) placed in a rotating wheel in front of the PSA.

The choice of the optimal polarization states for both PSG and PSA was governed by the minimization of measurement error propagation.^36^ It was shown that it requires the points representing the polarization states of PSG and PSA on the Poincaré sphere to be the vertices of the platonic solids (a regular tetrahedral in our case).^37^

### 2.2 Histopathological Labels and Mapping

**Fig. 2:**
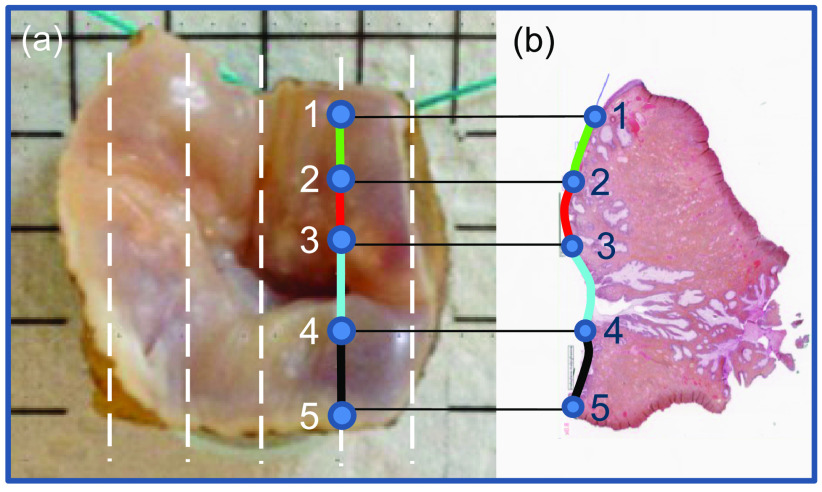
Illustration of the histological labeling of a cervical specimen; (a) color photo of a sample with the positions of cuts shown by the white dashed lines (see text). The sample was placed on a glass support graded with a step of 5 mm; (b) thin hematoxylin-eosin stained histological section with the color-coded annotated segments of cervical tissue epithelium (healthy, green; CIN3, red; glandular epithelium, cyan; not annotated, black).

The specimens of uterine cervix from 23 patients with histologically confirmed high grade cervical intraepithelial neoplasia (CIN3 or precancer) were collected in the Kremlin-Bicetre (KB) University Hospital, France. An original method was developed to produce detailed histological mapping superimposed on Mueller polarimetric images.^31^ This method consists of the following steps: (i) all samples are formalin-fixed (according to standard protocol); (ii) shallow parallel incisions at ∼3  mm separation distance from each other are made by a pathologist for each specimen before polarimetric imaging; (iii) the MM images of cervical specimens are recorded with the wide-field imaging Mueller polarimeter described in previous section; (iv) all cervical specimens are cut by a pathologist into the thick slices along the pre-cut lines as shown in Fig. 2(a); (v) thin histological sections (∼10  μm) are prepared from each slide using a microtome; (vi) the histological sections are annotated by a pathologist as shown in Fig. 2(b); and (vii) the diagnosis obtained on each histological section is repositioned along the pre-cuts on the polarimetric images.

**Fig. 3:**
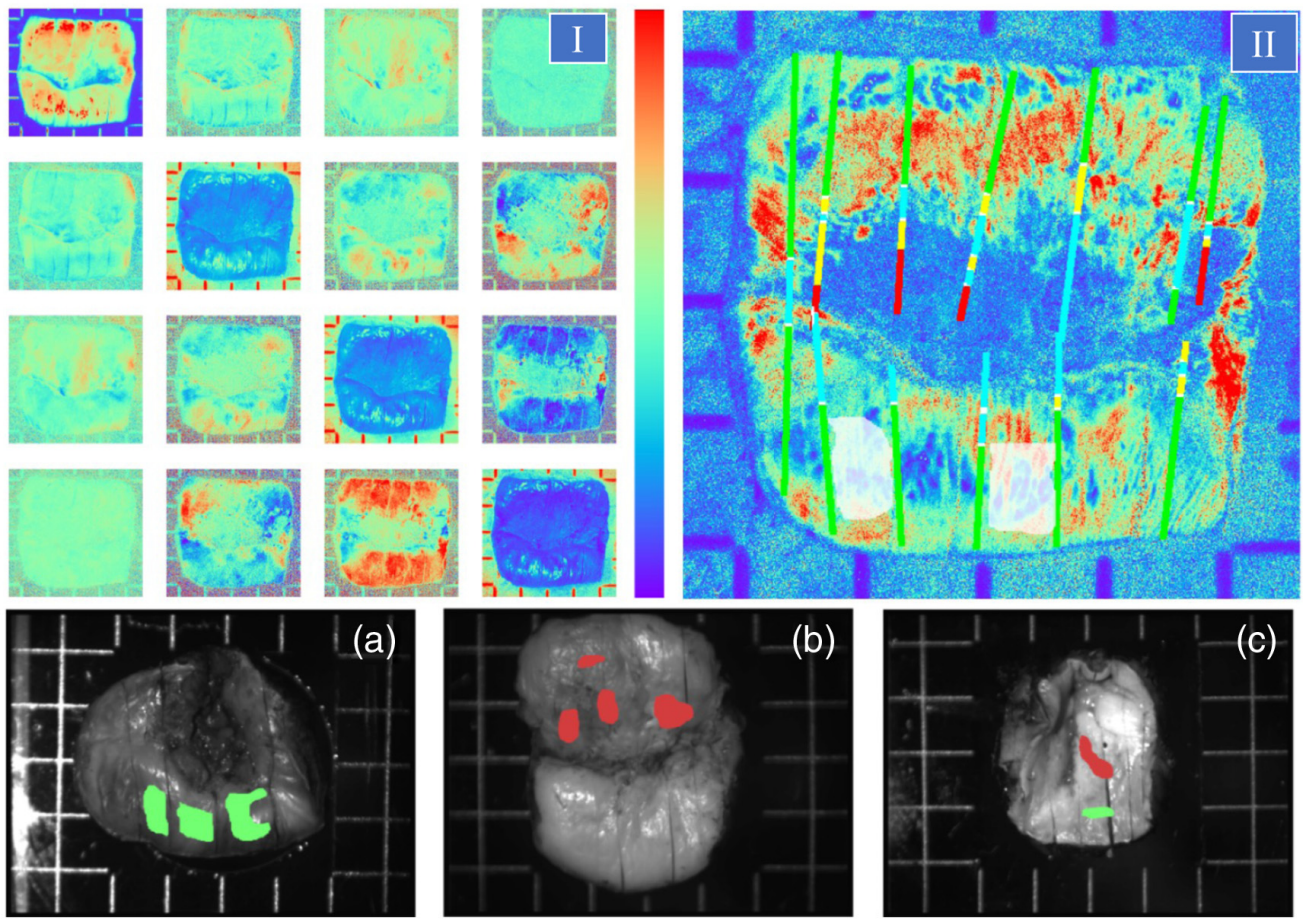
Example of experimental data with histological labeling with the actual size of each image being 3×3  cm; (I) 16 MM images of a sample. All MM images except $M_{00}$ are normalized by $M_{00}$ pixel-wise. With respect to the color bar, images on the diagonal range between 0 and 1, and the off-diagonal images range between −0.1 to 0.1; (II) the cut lines labeled with color-coded histological diagnosis (healthy, green; low grade CIN, yellow; high grade CIN3, red; glandular epithelium, cyan; metaplastic squamous epithelium, blue; not annotated, black) are superimposed on the scalar linear retardance image (in degrees). The semi-transparent white shapes overlaying the image illustrate how masked regions are selected between the adjacent cut lines labeled with the same histological diagnosis (see text). With respect to the color bar, this image ranges from 0 deg to 50 deg. The bottom row depicts examples of the intensity images for three specimens with the superimposed masks of histological diagnosis: (a) specimen with the healthy zones only, (b) specimen with the CIN3 zones only, and (c) specimen with both healthy and CIN3 zones. Pre-cancerous (CIN3) zones are rendered in red, and healthy zones are in green (scheme adapted from Ref. 33).

An example of the recorded MM images of a cervical specimen is shown in Fig. 3, (i). The experimental MM images were post-processed using the Lu-Chipman decomposition algorithm.^29^ The corresponding map of scalar retardance with the histology analysis placed along the cut lines is shown in Fig. 3, (ii). It was found that the projection of histologically labeled lines onto the polarimetric images tended to result in small vertical and horizontal displacement errors that may affect the accuracy of polarimetric diagnosis.^31^ To mitigate this effect and to increase the number of labeled polarimetric data, we manually selected the zones that are located between the segments of two adjacent cut lines tagged with the same histology diagnosis [e.g., white-dashed zones between the adjacent gray-colored segments of cut lines in Fig. 3, (ii)] and labeled all pixels of these zones with the same histology diagnosis. This approach was used to create binary masks for both healthy and CIN3 zones for all cervical specimens (shown in Fig. 3, bottom row). Due to the lack of histologically confirmed information for low grade lesions CIN1 and CIN2, we focused our efforts on a binary classification problem (CIN3 versus healthy tissue) using a set of labeled data.

Previous research has investigated the utility of Mueller polarimetry in the context of detecting pre-cancer in cervical tissue specimens. Rehbinder et al.^31^ obtained an average value of 83% for both sensitivity and specificity. Their work involved using the scalar retardance values calculated from the MM images of 17 formalin-fixed cervical specimens as a decision variable. The work undertaken by Kupinski et al.^33^ found that using the scalar retardance values or three smallest eigenvalues of the coherency matrix (4×4 complex-valued Hermitian semi-definite matrix^23^) permitted high performance, achieving AUC scores of 0.93 and 0.94, respectively. The high performance of depolarization metrics based on three smallest eigenvalues of the coherency matrix in discriminating between healthy and pre-cancerous regions was a novel finding because the detection performance of total depolarization from Lu-Chipman was close to that of a random classifier (AUC = 0.5). Using both the scalar retardance and the three smallest eigenvalues enabled an average AUC of 0.95. Interestingly, it was also established that only 6 of the 16 MM elements were needed to achieve such a performance. This indicates that not all MM elements are equally relevant to the pre-cancerous classification task. For instance, Heinrich et al.^34^ developed a general framework for the use of machine learning approaches in the classification of Mueller polarimetric data. This approach introduces a general metric, called empirical risk, allowing for training the different classifiers, choosing the polarimetric variables of interest, defining the hyperparameters of the classifiers, and comparing the performance of the classifiers. Nevertheless, it is now possible to extend beyond assessing the diagnostic performance of the scalar retardance and depolarization parameters. In particular, machine learning models can be trained directly on the labeled pixels of all images of the MM. The quality of these models’ predictions can be assessed by examining the overlap of the histologically labeled lines and the predicted classification zones.

In this paper, we utilize machine learning methods that require less overhead in their implementation. The first key difference is the manner in which model parameters, such as tree depth or number of neural network layers, are selected. The cross-validation scheme for model parameters implemented in Ref. 34 was not used due to the ability of our classifiers to achieve high performance regardless of changes to default parameter settings in this pre-screening task. Such an exhaustive search through the parameter space for optimal parameters incurs significantly greater computational cost and becomes a case of diminishing returns. In addition, we focus on the raw Mueller data instead of decomposed optical properties. This challenge is motivated by the desire to avoid pre-processing to reduce the computational effort. The approach by Heinrich et al. also used feature selection, reducing the raw data to “superpixels” to mitigate redundancy in the training process. The models that we chose to include in this work are less susceptible to data redundancy; thus this step could be omitted while retaining high performance, thereby optimizing the overall process based on the previously obtained insights.

### 2.3 Artificial Intelligence and Machine Learning Techniques

The underlying idea of ML is one of automating the process of building analytical models. Without explicit instruction, systems are required to learn from patterns in data to classify them or predict other information about them. In a biomedical context, this could be learning to predict whether a region of tissue is malignant or not. Supervised ML is a sub-category of ML that uses labeled data to train models.^38^ This means that a subset of data, or instances, is available that was labeled for the system to learn from, e.g., tissue regions labeled as malignant or not by a pathologist. After training, the resultant model is able to predict these labels when provided with unlabeled data. ML techniques can be used to solve a variety of tasks, one of which is classification wherein the system learns to map a vector into one of multiple classes.^39^ These classes are discrete labels, such as whether a tissue region is malignant or not in a binary task. There can also be more than two classes, e.g., grades of cancer. Another common task is regression, in which the model typically predicts continuous, numerical values instead of discrete labels.^40^ An example of this is quantifying biomarkers for disease states in tissues.^41^^–^^43^

In the aforementioned diagnostics context, each of the masked pixels taken from the 4×4 MM is treated as an instance in our dataset. Each instance has 16 features, with each feature corresponding to one of the 16 channels of the MM. Each channel contains different optical information about the pixel. Labels show the class to which a given pixel belongs: to the CIN3 class or the healthy class. In this work, a total of three ML classification techniques are used to carry out the task of predicting healthy and CIN3 masks in the cervical tissue section samples. Below, we provide the necessary background for these techniques including a decision tree (DT), multi-layer perceptron (MLP), and one-dimensional (1D) convolutional neural network (CNN).

**Fig. 4:**
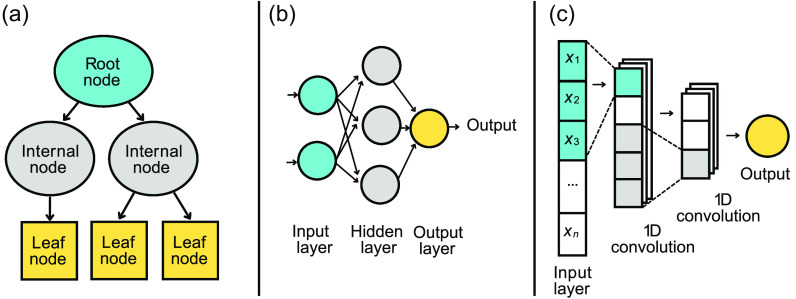
Schematic presentation of the three classification models used in this study: (a) DT, (b) MLP, and (c) 1D CNN.

DTs date back to the 1960s, when Morgan and Sonquist published their work on the first regression tree algorithm, AID.^44^ Following this, in 1972 Messenger and Mandell published the first classification tree algorithm, THAID.^45^ Breiman et al. combined the strengths of AID and THAID along with several extensions in their 1984 publication on classification and regression trees (CART), one of the most widely recognized DT algorithms to date.^46^ They are typically visualized as trees, with a simple example shown in Fig. 4(a). DTs split the dataset containing the pixels, through a series of choices, into the CIN3 and healthy classes.

The trees consist of nodes and branches, such that the root node and internal nodes represent the decisions, the branches (denoted by black arrows) are the outcomes of these decisions, and the leaf nodes contain the class labels.^47^ A decision could be whether a given pixel in a particular channel exceeds a threshold value, e.g., 0.5. Instances that have a value below 0.5 are sent down the left branch to one sub-node, and those above 0.5 will be sent down the right branch to the other sub-node. It is also possible to have non-binary decisions, such that a node maps into more than two sub-nodes. The algorithm chooses to include decisions in the tree that split the feature subspace such that instances with the same class labels are grouped together.^48^ In the context of this paper, this means that the pixels are recursively partitioned into increasingly homogeneous sub-nodes, by grouping CIN3 instances together and healthy instances together as much as possible.

To measure the homogeneity of the class labels, this typically involves minimizing entropy or the Gini index.^49^ These measures essentially state how impure or heterogeneous a collection of instances is with respect to each class label j in a given node i. For entropy this involves computing the Shannon entropy of the n classes:

$${\text{Entropy}}_{i}=-\overset{n}{\underset{j=1}{\sum }}p_{ij}\;\log_{2}\;p_{ij}.$$ (1)

In this work, n=2, which are the two class labels, CIN and healthy. Shannon entropy treats the class frequencies of the instances in each partition as a probability $p_{j}$. Computing the Gini index involves subtracting the sum of the squared probabilities (or proportions) of each class from 1:

$${\text{Gini}}_{i}=1-\overset{n}{\underset{j=1}{\sum }}p_{ij}^{2}.$$ (2)

MLP is another technique explored in this work. Perceptrons were initially developed as hardware by Frank Rosenblatt^50^ before their algorithmic implementation. They are linear classifiers, meaning they classify inputs by forming a weighted, linear combination yielding an output value or label. Perceptrons are the very basis of MLPs, which are comprised of many perceptrons. Further still, MLPs can be considered the precursor to larger neural networks and fall within an area of AI that aims to solve complex computational tasks by imitating human brain function. The perceptron (otherwise often referred to as a neuron) is the fundamental unit of an MLP or neural network and is shown as a circle in Fig. 4(b). In essence, neurons are computational units that manipulate a weighted input signal to produce an output signal using an activation function. The weights are analogous to the coefficients in a regression model. Each neuron also has a bias, which is similar to the intercept in regression. Activation functions are a function of the weighted inputs and the bias. In a binary classification task, a very simple implementation of this can be a step function in which, if the weighted input exceeds a threshold such as 0.5, the neuron will output a value of 1.0 and otherwise 0.0, with each corresponding to one of the two possible classes. Various other non-linear activation functions can represent more complex relationships between the inputs and output,^51^ e.g., “ReLU” or “LeakyRelu.” In terms of their overall architecture, MLPs are made up of an input layer, one or more hidden layers, and an output layer, each containing neurons. The key algorithm underpinning the success of MLPs is back-propagation, which comprises a forward pass and a backward pass.^52^ In the forward pass, the inputs are fed into the MLP with weights initialized to some set of starting values. Each hidden and output neuron operates on its inputs by multiplying them by the initial weights, summing the result, and passing this through a non-linear activation function to produce the predicted outputs. The difference between the true output and the predicted output of the output layer is used when calculating a loss function. During the backward pass, the resultant loss is used to adjust the weights such that the predicted output of the MLP is closer to the true output. When the activation function is differentiable, this update is typically carried out using an optimization algorithm such as gradient descent or “Adam,” which changes the weights in proportion to the negative of the derivative of the error term.^53^ In simple terms, the purpose of this optimization algorithm is to find the values for the weights that make the loss function as small as possible.

CNNs are a type of artificial neural network that use convolutional layers to extract local features from the data. Similarly to MLPs, CNNs draw their inspiration from the human brain. The structure or connectivity of a CNN is akin to that of the visual cortex.^54^ Each neuron responds to stimuli in one portion of the visual field at a time, known as the receptive field.^55^ A filter, equal in size to this receptive field, is element-wise multiplied with the pixels that it overlaps. This operation is known as a convolution.

Convolutional layers containing this type of processing are able to capture spatial and temporal dependencies between features through the use of filters. As shown in Fig. 4(c), a convolution layer typically uses a plurality of filters and produces multiple feature maps, each with a reduced number of features. With increasing depth, the CNN refines the number of features to those that are highly relevant to the classification task. Typically, early layers in a CNN are dedicated to extracting low-level features such as colors and edges, whereas later layers are responsible for learning higher level, holistic features of an image. Reduction of the size of the feature maps (down-sampling) is obtained either by a suitable stride of the convolution or by means of a pooling layer. Unlike convolutional layers, which contain learnable weights, pooling layers simply apply a given function. At the output of the CNN, a flattening layer can be used to transform a feature map into a 1D vector. This vector can then be classified using a linear weighting and an activation function, similar to that described in MLPs.

## 3 Capture-to-Classification Pipeline

Recent progress and wide adaptation of artificial intelligence and machine learning (AIML) techniques capable of solving complex, real-world problems make them a highly desirable tool in the field of biomedical optical imaging and biophotonics. Success has been demonstrated with integrating ML techniques with hyperspectral imaging (I) of human skin, resulting in pioneering applications in 3D computer graphics, optical sensing, and imaging.^56^^–^^59^

**Fig. 5:**
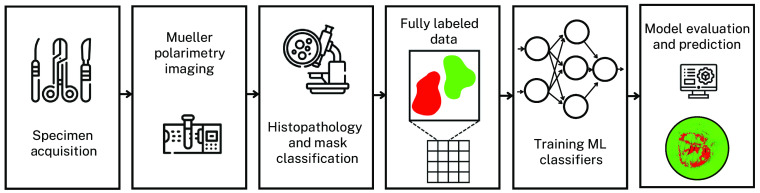
Schematic presentation of the capture-to-classification pipeline developed in house. Acquired specimens are measured with imaging Mueller polarimeter and undergo histopathological classification. Subsequently a labeled dataset is created within tagged regions of healthy and CIN3 tissues. Several ML methods are trained, and their corresponding accuracies are compared.

One context of particular interest involves finding features that are characteristic of diseased tissue, i.e., cancer, as these are not always evident when pathologists examine a tissue sample. In this work, we developed a specialized capture-to-classification pipeline (shown in Fig. 5) that utilizes the expertise of pathologists and aims to assist with automated, real-time, *in situ* classification of cervical tissue samples.

### 3.1 Data Collection, Pre-Processing, and Quality Tests

MM images of the cervical specimens were acquired at several wavelengths (450, 550, and 600 nm, image resolution 600×800  pixels). Each of the 16 channels contains different polarization information. We did not apply any algorithms of the non-linear data compression (i. e., the decomposition algorithms) and used the recorded MM data to construct two broad datasets. The first one uses the four diagonal elements of MM, and another one uses all 16 values. It is known that, in general, bulk biological tissues demonstrate strong depolarization, moderate linear birefringence, and negligible linear diattenuation.^20^ In the absence of the anisotropy of the optical refractive index, the depolarization properties of tissue can be estimated using the values of the last three diagonal elements of its MM.^60^ Otherwise, these values can be considered to be a fused metric for both effects, namely, depolarization and linear retardance with the element $M_{00}$ representing the total reflectivity of tissue. The decision to use two different formats of the dataset is dictated by the necessity to ascertain whether the contribution to performance of the off-diagonal elements is sufficient to justify their inclusion. It is speculated that any additional accuracy that they can provide does not offset the computational cost. This is a method of feature selection, wherein features that are not relevant to the classification task or are redundant in that they do not add additional predictive performance from other features are discarded. Therefore, it improves the computation time by minimizing the model complexity.^61^

Using the histopathology masks described in Sec. 2.2, we extracted the pixels from the MM images that are known to belong to either CIN3 or healthy zones and labeled these pixels accordingly. We did not consider the low grade CIN, glandular epithelium, and metaplastic squamous epithelium because there was an insufficient number of pixels labeled with each of these histology conditions across the images. As a first step, we restrict ourselves to binary classification, focusing on the correct detection of CIN3 zones, because such lesions require surgical intervention, whereas for other pathological conditions, watchful waiting is recommended.^7^

The unlabeled pixels were not included in the training process. This extraction yielded 57,925 samples of CIN3 pixels and 133,910 healthy pixels, across all cervical tissue samples. Each instance has either four features or 16 features depending on which of the broader aforementioned datasets is used. The ratio between CIN3 and healthy instances translates to roughly 30% of the data belonging to CIN3 (class 1) and 70% belonging to healthy (class 2). Because there are high numbers of instances in both classes, the class imbalance problem is avoided, and there is minimal risk of model bias.

### 3.2 Data Quality Assessment

**Fig. 6:**
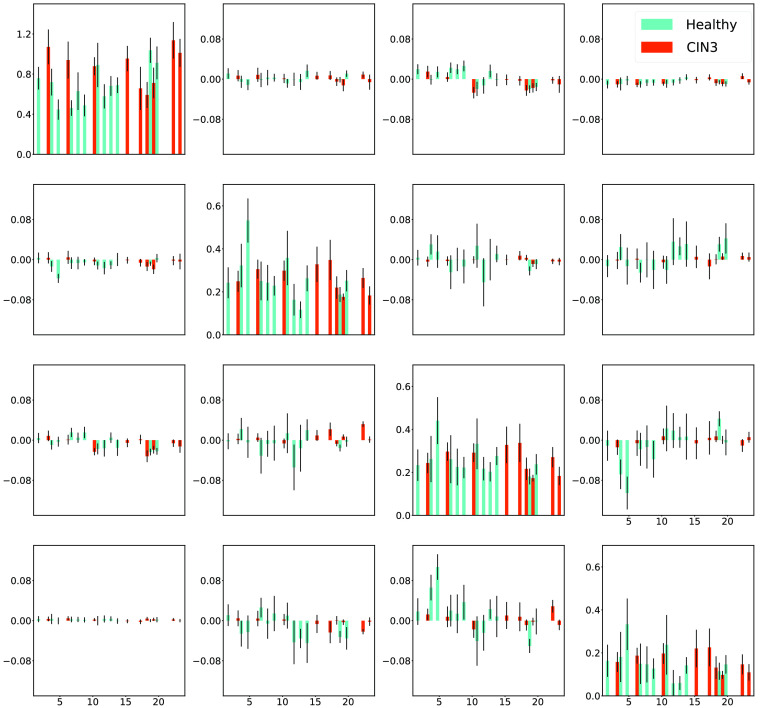
Mean value and standard deviation of the distributions of labeled pixels in the images of 16 elements of MM for all 23 specimens measured at 550 nm. Standard deviations are denoted by black bars, and the horizontal axis corresponds to the sample ID number.

The extracted pixels belonging to each of the two classes then underwent a series of tests. The first test explores whether the samples belonging to each of the two classes can reliably be considered to come from the same distributions. This can provide assurance that there is minimal variability in the samples that is unrelated to the classification task itself, i.e., confounding factors. To determine whether the samples came from the same distribution, a Kruskal–Willis test is applied to the pixels labeled “CIN3” and the pixels labeled “healthy” independently. The Kruskal–Willis test reveals that the instances belonging to each class do indeed follow the same distributions as each other, respectively. Their distributions in terms of mean and standard deviation are plotted in Fig. 6. It is worth noting the scale of the individual elements differences as the diagonal elements vary across a larger range than the non-diagonal elements.

We do not expect the MM elements to have normal distributions; to validate this and build highly tailored classifiers, normality tests were undertaken. Due to the large size of the dataset used in this work, the Kolmogorov–Smirnov test is used in favor of the more conventional, Shapiro–Wilk test for normality, as the latter is not well-suited for sample sizes containing more than 2000 instances.^62^ It was established that all samples are not normally distributed according to the Kolmogorov–Smirnov test, which concurs with our expectations.

### 3.3 Testing and Training Sets

Two approaches for dividing the instances between training and test sets were considered in this work. The first approach involves a uniform, stratified, shuffled split in which 10% of instances are assigned to the test set and the remaining 90% form the training set. This approach is conventional; however, it is limited when entire samples are predicted. For instance, when the model learns from pixels belonging to all samples by nature of a shuffled split, the measured performance becomes an over-estimate of true performance. To address this, a second, less commonly used splitting approach is considered. This is known as leave-one-out cross-validation, wherein the model is trained a total of 24 times, with each sample taking a turn as the test set and the remainder constituting the training set. This will lead to a poorer but more accurate performance than the previous approach as it does not predict on samples that were used to train the model.

### 3.4 Implementation and Evaluation of AIML Methods

The purpose of this work is to obtain an ML classifier with high sensitivity and specificity in distinguishing pre-cancerous and healthy regions of cervical tissue sections. To accomplish this task, three different ML models were trained and their performance evaluated. These ML approaches include a DT, multilayer perceptron (MLP), and 1D CNN. The implementation of these approaches involved the use of Python libraries, such as Scikit-learn, Keras, and Tensorflow. The optimal parameter settings for each ML method were obtained through empirical search and are described below.

Our DT implementation uses CART and splitting occurs according to the Gini index [see Eq. (1)], as opposed to Shannon entropy. Although both have comparable performance,^63^ the former method is less computationally expensive than the latter due to the logarithm calculation [Eq. (2)]. A maximum tree depth of 10 is used to limit the tree depth. There are various motivations for this. One includes that it reduces model complexity, such that the computational time is reasonable. Allowing a model to contain many layers, such that it is highly complex, can result in diminishing returns whereby the performance does not increase significantly but the training time is very high. Furthermore, a phenomenon known as over-fitting can occur. This arises when the model becomes too specific to the training data, such that it achieves very high performance on training data, but cannot generalize well to test data and consequently cannot achieve high test performance. Finally, models that have limited depth are more comprehensible than those without restrictions, by nature of there being many fewer nodes or decisions.

The architecture of our MLP consists of three hidden layers, each with 32 hidden nodes. Following a similar tangent that motivated the design of the DT used in this work, the choice of this architecture for the MLP primarily stems from a desire to balance performance and complexity. If the number of hidden nodes and layers is insufficient, the MLP will risk under-fitting the data due to its lack of complexity, such that it is unable to map the relationships between the features, or channels, and the class labels. If this number is instead too high, the increase in complexity will not necessarily correspond to a proportionate increase in performance and may instead be deleterious, resulting in a worse performance due to over-fitting behavior. Thus, it is a matter of determining the best balance of hidden layers and nodes. We used the rectified linear unit (ReLU) as the activation function,^64^ for it is widely recognized that non-linear functions are able to capture complex relationships that linear activation functions can fail to describe. The Adam optimization algorithm is used for changing the weights according to the cross entropy loss function. It has been demonstrated that Adam optimization is able to outperform conventional optimization methods, e.g., gradient descent,^65^ and cross-entropy is used as the loss function due to its known utility in classification tasks. The training process is allowed to run for up to 100 iterations or weight updates. Early stopping is implemented, however, such that if the performance does not improve by 0.1% between iterations, the training process is terminated to help minimize the risk of over-fitting.

Our architecture of the 1D CNN encompasses one hidden layer containing 16 filters (corresponding to the number of channels), a kernel size of 4, and similar padding. In addition to this, an ReLU activation function, an Adam Optimizer, and a cross-entropy loss function were employed. The training process was allowed to run for 100 epochs, with no early stopping criteria. The application of a CNN in this manner is recognized as somewhat unconventional as typically a CNN operates on a region of pixels instead of one pixel at a time. This means of using a CNN was motivated by the need to make fair comparisons between the different machine learning models and examine whether the 16 channels for a given pixel contain enough information independent of other pixels to be able to classify it.

To evaluate each of the three ML models in their ability to predict pre-cancerous and healthy masks, several evaluation metrics were recorded and analyzed. These metrics include area under the curve (AUC) or accuracy, sensitivity, specificity, and training time. AUC is used for 90:10 train–test split, and accuracy is used for leave-one-out cross-validation. AUC is not applicable for the latter due to the nature of the splitting approach, wherein only three samples have both positive and negative labels, upon which the metric relies. Such an evaluation process pertains to the difference between the true mask values for each pixel and those mask values predicted by the ML models. Two averaging approaches are used. In one, 90% of the data is used for training with the remaining 10% withheld for testing or evaluation purposes. The second approach involves using 23 samples for training, with one being withheld for evaluation, in a leave-one-out cross-validation process. In both approaches, the results were averaged over many runs, with the former using the average of 30 different random seeds and the latter corresponding to the average of a different sample being withheld as the test set each time. An additional evaluation procedure was also explored, whereupon entire samples are predicted. All models were trained using a 2.8 GHz quad-core Intel Core i7 processor.

## 4 Results and Discussion

The performances of the two splitting approaches across three ML models are given in Table 1. The results were averaged for each approach, such that each cell contains the mean ± standard deviation. The overall results from the 90:10 training:test split approach are better than those obtained using leave-one-out cross-validation. This is as anticipated, as the former approach trains on pixels across all samples. This means that the composition of the training and test sets, after splitting the original dataset, will likely be very similar. Conversely, in the latter approach, in which one sample is withheld as the test set, it is plausible that the remaining samples used for training may fail to contain all information necessary to obtain a high performance on the withheld test set as this test set may contain some unique information. This more accurately reflects a clinical setting, in which the model will be used to classify a completely unseen sample, not used in training. Therefore, it can be argued that the 90:10 training:test split approach over-estimates the performance of a model, and the leave-one-out cross-validation approach or similar should be applied to obtain a more realistic model evaluation. It was postulated that the reduced performance of the leave-one-out cross-validation approach may be offset by there being relatively more data available for training, with 22 out of 23 samples being used to train as opposed to just 90% of the data for any given training iteration. Furthermore, exploratory data analysis revealed that the 23 samples were found to follow similar distributions to each other. As a result, the leave-one-out cross-validation approach still manages to achieve an accuracy of 0.812±0.21 for the best model, the 1D CNN.

**Table 1:** Comparison of average performance across the three ML models (DT, MLP, and 1D CNN) using two different approaches for train–test split strategies. The results reported are the mean value of the corresponding column header ± standard deviation value.

	AUC/accuracy	Specificity	Sensitivity	Training time (s)
90:10 train–test split with shuffled stratified sampling				
DT	0.962 ± 0.00	0.977 ± 0.00	0.946 ± 0.00	3.585 ± 0.06
MLP	0.986 ± 0.00	0.991 ± 0.00	0.981 ± 0.01	13.132 ± 2.29
D CNN	0.964 ± 0.00	0.978 ± 0.00	0.952 ± 0.01	268.777 ± 116.3
Leave-one-out cross-validation				
DT	0.762 ± 0.20	0.822 ± 0.16	0.697 ± 0.23	3.729 ± 0.27
MLP	0.803 ± 0.23	0.846 ± 0.22	0.756 ± 0.25	16.594 ± 9.6
D CNN	0.812 ± 0.21	0.851 ± 0.20	0.763 ± 0.25	329.939 ± 171.03

In the 90:10 splitting approach, the MLP has the best AUC value of 0.986±0.00. The 1D CNN model is the next best with an AUC score of 0.964±0.00. The baseline method, DT, has an AUC score of 0.962±0.00. The DT is limited in that it can only separate the instances in a linear fashion. It is likely that the 1D CNN is unable to surpass the performance of the MLP as it was implemented in a per-pixel classification task; thus it cannot take full advantage of the convolutional layers. The specificity of the best performing model, the MLP, is better than the sensitivity. Interestingly, this pattern is also observed in the other two ML models. This suggests that it is an easier task to classify healthy pixels than it is to classify CIN3 pixels. This finding is not surprising as the majority of the pixels, ∼70%, in the dataset used for this work correspond to healthy cervical tissue. Because there are more healthy instances to learn from, it is therefore expected that an ML model will achieve a better performance predicting healthy pixels than CIN3 pixels.

The DT has the fastest training time of ∼3.5  s in both approaches. The MLP models then take about around 13 and 16 s to train in the leave-one-out and 90:10 splitting approaches, respectively. The 1D CNNs take around four to five min in each approach. Therefore, the MLP could be considered the best in terms of performance and computational time, in this per-pixel classification task. Although, because training only needs to take place once, it is more relevant that the inference time be lower than that of training. At inference time, the three models all take on the order of seconds to classify a given sample. The training times are presented to highlight the trade-off between marginal improvements in performance and substantial increases in computational time. The standard deviations of the 90:10 splitting approach are very small for the performance metrics, indicating that the ML models were able to consistently converge on high performing solutions, likely due to the fact that approach permits a similar composition for the training and test sets. The same cannot be said for the leave-one-out approach in which the standard deviations are relatively large. This again highlights the need for more data as it is evident that not all information necessary to gain a high predictive performance on a withheld sample can be obtained from the other samples used for training.

**Fig. 7:**
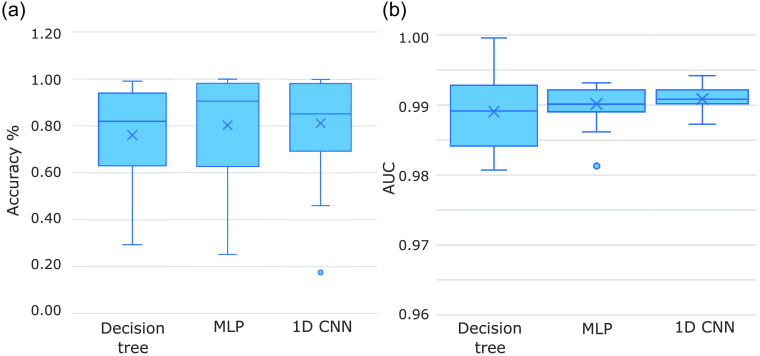
Distributions for leave-one-out cross-validation accuracy values, over the 23 samples for (a) all models and 90:10 train:test split AUC values, (b) over the 30 random seeds.

The distribution of AUC performance across the seeds is relatively narrow for the 90:10 training:test split approach. This is a reasonable result as the approach involves taking data from all samples, randomly shuffling, and using 90% of this shuffled data for training (see Fig. 7). Thus, both the training and test sets are expected to have a similar composition. The variation in the results is much smaller than that in the leave-one-out cross-validation approach because of this similarity in composition. Examining the performance on a per-sample basis for leave-one-out cross-validation reveals that some of the models for some samples perform very well as the maximum AUC score is ∼1.0 in each case. The model of the sample with the worst performance, however, has an AUC score of <0.2. This indicates that, when the data are split on a per sample basis, there are trends in the data that are not captured by other samples. This suggests that, to train a model that is capable of diagnosing a completely unseen tissue section, it would be necessary to learn from many more than 23 samples to confidently capture all of the unique features.

**Fig. 8:**
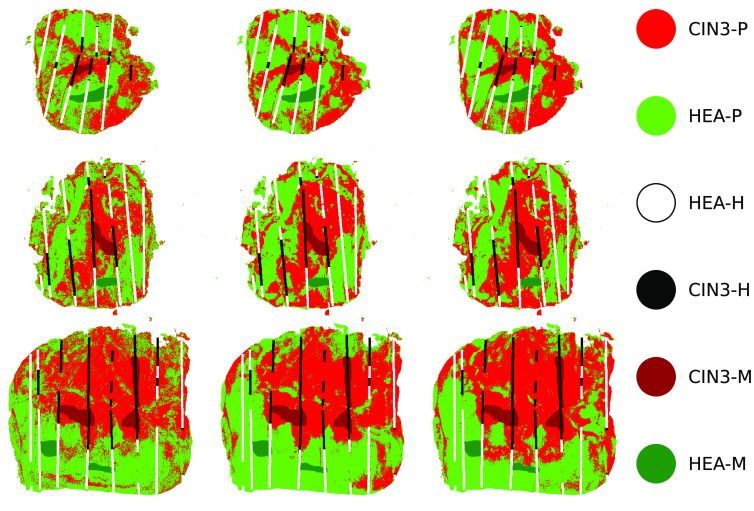
Whole specimen prediction across three samples (columns) and the three techniques (rows). From left to right, the sample IDs are 19, 20, and 23. From top to bottom, the techniques are DT, MLP, and 1D CNN. In each image, the whole specimen predictions are shown, indicated by CIN3-P and HEA-P. In addition, the zone masks described in Sec. 2 are shown by CIN3-M and HEA-M. Finally, the overlap of cut lines annotated by histopathology analysis with CIN3-H and HEA-H is used as a metric for accuracy in whole sample prediction.

It is worth noting that sensitivity and specificity are on average quite low for the leave-one-out cross-validation approach, particularly the latter. These metrics are based on true positive and true negative rates, thus suggesting that for a given sample the rate of calling negatives correctly or positives correctly is limited. Therefore this again demonstrates that not all of the data necessary for predicting a given sample are available in other samples, highlighting the need for a large dataset for training. Due to the significant variation in the diagonal elements relative to the off-diagonal elements, as shown in Fig. 6, we initially hypothesized that it might be sufficient to only retain the former for model training. However, during our preliminary work, we discovered that such an alternative design led to a notable decrease in performance (up to 10% to 20%) when we excluded the off-diagonal elements, necessitating a more complex ML structure. Consequently, we decided to use the full information, which is pertinent to the task of predicting CIN3 in cervical tissue sections and is contained in both the diagonal and off-diagonal elements of the MM. We also performed qualitative testing by carrying out whole sample prediction. Figure 8 shows that there is a consensus across the three techniques for where the healthy regions lie (bright green) and where the CIN3 regions are (bright red). It seems that the DT model, unlike the two neural network techniques, has a noisier classification. This is evident from the less defined boundaries where there is a mixture of CIN3 and healthy pixels in large regions beside the central CIN3 region. In general, the histopathology lines do overlap with the whole sample predictions across the techniques. It does, however, appear that the further from the mask the predictions are, the poorer the histopathology lines overlap with these regions. Regions that are difficult for a pathologist to assign a mask likely have some tissue properties in common. With this line of reasoning that masked pixels are potentially more similar to each other than those unable to be assigned a mask, this could consequently make it a harder task for a model to classify areas further away from the masked regions. This further supports the incentive for capturing more data to yield high performing predictive models. High performance is shown to be achievable with more data from the fact that the closer the whole sample predictions are to the masks, the better the overlap with the histopathological lines is. Particularly in the last sample (bottom row of Fig. 8), the whole sample prediction appears more accurate in terms of overlap of the histopathology lines.

## 5 Conclusions and Future Work

This work showcases the synergy of combining Mueller polarimetry and ML techniques to undertake the task of screening for pre-cancerous conditions in cervical tissue sections. We have undertaken an initial investigation into applying ML methods in this particular context as a perspective application. We demonstrated the ability of both simpler and more advanced ML methods to achieve high performance in the per-pixel classification task in a matter of seconds. The evaluation also encompassed whole sample prediction in which prediction was no longer limited to masks with known labels to compare against. Instead, histopathological mapping was used to qualitatively infer and assess whole sample prediction. Furthermore, to robustly measure model performance, we averaged results through two approaches: a 90:10 training:test set splitting approach and a leave-one-out cross-validation approach, with the former being a more conventional process and the latter serving as a more conservative approach in that the whole model prediction was subject to less bias, as none of these pixels were used for training. Our future work directions include developing a more sophisticated approach for inferring the accuracy of whole sample predictions than a qualitative or visual assessment using the superimposed histopathological lines and polarimetric maps of a specimen and testing new design of polarimetric instruments including partial Mueller polarimetry^66^ and utilization of the light’s orbital angular momentum.^67^ In addition, an assessment of the pathologists’ uncertainty relative to these histopathological lines using Bayesian statistics methods is also of particular research interest. This will involve generating probability distributions of classifications, as opposed to point estimates, so information on model confidence is available alongside the predictions.

## Code, Data, and Materials Availability

Code and data are available upon reasonable request.
